# A randomized, placebo-controlled experimental medicine study of RIPK1 inhibitor GSK2982772 in patients with moderate to severe rheumatoid arthritis

**DOI:** 10.1186/s13075-021-02468-0

**Published:** 2021-03-16

**Authors:** Kathleen Weisel, Scott Berger, Katie Thorn, Peter C. Taylor, Charles Peterfy, Hilary Siddall, Debra Tompson, Susanne Wang, Emilia Quattrocchi, Susan W. Burriss, Jochen Walter, Paul Peter Tak

**Affiliations:** 1 GlaxoSmithKline, Collegeville, PA USA; 2 GlaxoSmithKline, Stockley Park, Uxbridge, UK; 3 Botnar Research Centre, Nuffield Department of Orthopaedics, Rheumatology and Musculoskeletal Sciences, University of Oxford, Oxford, UK; 4 Spire Sciences, Inc., Boca Raton, FL USA; 5 GlaxoSmithKline Medicine Research Center, Gunnels Wood Road, Stevenage, Hertfordshire SG1 2NY UK; 6 Rheumatologische Schwerpunktpraxis, Rendsburg, Germany; 7 Present address: Cambridge University, Cambridge, UK; 8 Present address: Ghent University, Ghent, Belgium

**Keywords:** Pharmacokinetics, Pharmacodynamics, Receptor-interacting protein kinase 1, RIPK1, Rheumatoid arthritis

## Abstract

**Background:**

Receptor-interacting protein kinase 1 (RIPK1) is a key mediator of inflammation through cell death and proinflammatory cytokine production. This multicenter, randomized, double-blind (sponsor-unblinded), placebo-controlled, experimental medicine study evaluated the safety, pharmacokinetics (PK), and preliminary efficacy of GSK2982772, a RIPK1 inhibitor, in moderate to severe rheumatoid arthritis (RA).

**Methods:**

Patients with moderate to severe RA who had received ≥12 weeks’ stable-dose conventional synthetic disease-modifying antirheumatic drug (csDMARD) therapy were randomized (2:1) to GSK2982772 60 mg or placebo orally 2 or 3 times daily for 84 days. Safety, PK, disease activity, joint damage, and pharmacodynamic (PD) biomarkers were assessed at days 43 and 85.

**Results:**

A total of 52 patients were randomized (placebo, 18; GSK2982772, 34). Adverse events (AEs) were reported in 13 (72%) in patients in the placebo group (*n* = 3 b.i.d; *n* = 10 t.i.d.) and 20 (61%) in the GSK2982772 group (*n* = 3 b.i.d; *n* = 17 t.i.d.). All treatment-related AEs were mild/moderate, except one severe case of alopecia areata at day 49 and retinal vein thrombosis at day 66 (which led to withdrawal from the study) in patients receiving GSK2982772 t.i.d. Disease Activity Score in 28 Joints–C-reactive protein (DAS28-CRP) scores, ACR20/50/70 response, and rates of low disease activity and remission were similar between placebo and GSK2982772 arms.

**Conclusions:**

These results suggest that inhibition of RIPK1 activity at the GSK2982772 exposure levels evaluated do not translate into meaningful clinical improvement of RA.

**Trial registration:**

ClinicalTrials.gov Identifier: NCT02858492. Registered 8 August 2016.

**Supplementary Information:**

The online version contains supplementary material available at 10.1186/s13075-021-02468-0.

## Introduction

Rheumatoid arthritis (RA) is a chronic immune-mediated inflammatory disease that affects synovial tissue in multiple joints [1]. Despite available treatments, there is still a significant unmet need for safe and tolerable therapies, particularly in an oral formulation, that lead to improved rates of clinical remission and increased physical function in patients with moderate to severe RA [2, 3].

Central to the pathogenesis of RA are interactions between innate immune cells (monocytes, dendritic cells, mast cells) and adaptive immune cells (CD4+ T cells, B cells, plasma cells) targeting the synovial membrane [1, 4]. Tumor necrosis factor (TNF) is a key cytokine that drives chronic inflammation in RA [5, 6]. Binding of TNF to its receptor, TNFR1, results in one of three potential cellular outcomes: NF-κB activation, apoptosis, or necrosis. NF-κB activation by agents such as pristine and streptococcal cell wall have been associated with induction of rat arthritis [7, 8], and mice with myeloid cell-specific deficiency of A20, a deubiquitinase negatively regulating NF-κB signaling, spontaneously develop polyarthritis with typical features of RA [9].

RIPK1 blockade provides a potential new therapeutic approach for immune-mediated inflammatory diseases. RIPK1 is a critical kinase regulator of cell death (apoptosis and necroptosis) and proinflammatory cytokine production downstream of numerous pathways and signaling receptors, including the TNF family of cytokines, ligands for toll-like receptor (TLR) 3/TLR4, and interferons [10]. Cell death by necroptosis is highly inflammatory due to the release of cellular contents and the subsequent activation of pattern recognition receptor signaling and innate immunity. RIP1 has dual roles as a kinase and a scaffolding protein and serves as an upstream checkpoint for both cell death and survival [10]. The scaffolding function facilitates other immune processes including TNF-mediated classical apoptosis and NF-κB signaling [10–12].

RIPK1 inhibitors prevent TNF-dependent inflammation in multiple preclinical models (TNF shock, SHARPIN) [13, 14]. Mutations of HOIL, HOIP, or NEMO, proteins in the linear ubiquitination chain assembly complex, have been linked to activation of RIPK1 kinase activity and result in TNF-dependent auto-inflammation [10, 15, 16]; similar mutations in mice also result in inflammatory phenotypes that can be blocked with RIPK1 inhibitors [17]. In RA patients, expression of TNF and its signaling intermediates, including RIPK1, have been shown to be constitutively increased in peripheral blood mononuclear cells compared to healthy controls [18].

RIPK1-mediated necroptosis has also been implicated in increased cytokine production and inflammatory diseases [19–21]. Inhibition of RIPK1 blocked necroptosis, suppressed osteoclastogenesis, reduced synovial expression of proinflammatory cytokines, and decreased arthritis progression in a collagen-induced mouse model of RA [22].

GSK2982772 (5-Benzyl-N-[(3S)-5-methyl-4-oxo-2,3-dihydro-1,5-benzoxazepin-3-yl]-1H-1,2,4-triazole-3-carboxamide) (for chemical structure, see Additional file 1 Figure S1) is an oral selective inhibitor of RIPK1 that binds an allosteric pocket of the RIPK1 domain and thus inhibits RIPK1-mediated cell death and cytokine production in preclinical models [23], and its safety and tolerability has been demonstrated at doses up to 120 mg b.i.d. in healthy volunteers [24]. The current experimental medicine study examined the safety, tolerability, pharmacokinetics (PK), pharmacodynamics (PD), and preliminary efficacy of GSK2982772 following 12 weeks of treatment in patients with moderate to severe RA.

## Methods

### Study design

This multicenter, randomized, double-blind (sponsor-unblinded), placebo-controlled experimental medicine study was performed between October 17, 2016, and October 22, 2018, in patients with moderate to severe RA at 12 study centers in five countries (Germany, Italy, Poland, Russia, and Spain). Participants were initially assigned to either GSK2982772 60 mg orally b.i.d. or placebo b.i.d. Following a protocol amendment, participants were randomized to GSK2982772 60 mg orally t.i.d. or placebo t.i.d.

The primary objective was to assess the safety and tolerability of GSK2982772. Secondary objectives were to quantify the plasma concentrations of GSK2982772 following administration 60 mg b.i.d. and t.i.d. In addition, the impact of GSK2982772 on bone, cartilage, and synovial parameters (e.g., bone erosion, synovitis, osteitis, and joint space narrowing); inflammatory biomarkers; and clinical disease activity was investigated. The study was approved by the ethics committee/institutional review board at every participating institution and was conducted according to the recommendations of Good Clinical Practice and the Declaration of Helsinki. All patients provided written informed consent to participate in the study.

Within 30 days of screening, eligible patients entered an 84-day treatment period, which was followed by a 28-day follow-up period. Patients were randomly assigned in a 2:1 allocation ratio to receive either oral GSK2982772 60 mg or placebo (b.i.d. per the initial protocol or t.i.d. following approval of a protocol amendment in all countries) for 84 days. Randomization to treatment groups was via a central Interactive Technology System. The randomization number was assigned from a randomization schedule generated prior to the start of the study, using validated internal software. A data review committee (DRC) of the sponsor was unblinded for routine pharmacovigilance and internal decision-making purposes regarding other studies with GSK2982772. Informal review of DAS28-CRP data (and inflammatory biomarkers and other endpoints if available) was conducted by the DRC after an appropriate number of subjects completed day 43 (week 6). The formal interim analysis, to assess futility as planned per the protocol, was not conducted based on the recommendation by the DRC because, due to increased speed of recruitment, all subjects had completed the treatment phase before there was sufficient data to perform a futility assessment. An internal GSK Safety Review Team (SRT) also reviewed blinded safety data, including clinical laboratory parameters and adverse events, at appropriate intervals during the period of study conduct. Treatment codes could be unblinded by the investigator or treating physician only in the case of a medical emergency or in the event of a serious medical condition, when knowledge of the investigational product was essential for the clinical management or welfare of the patient. GSK Global Clinical Safety and Pharmacovigilance (GCSP) staff could unblind treatment codes in the event of a serious adverse event (SAE).

### Study population

Patients were enrolled if they were between 18 and 75 years old, had a confirmed diagnosis of RA according to the revised 2010 American College of Rheumatology (ACR)/European League Against Rheumatism (EULAR) classification criteria [25], and had a disease duration of ≥12 weeks. Eligible patients had moderate to severe RA as indicated by a swollen joint count ≥4 (28-joint count), tender joint count ≥4 (28-joint count), and Disease Activity Score in 28 Joints–C-reactive protein (DAS28-CRP) ≥ 3.2 and CRP ≥ 5.0 mg/L at screening. Additional inclusion criteria included naive status for any biologic therapy for RA (previous exposure to a single anti-TNF biologic was allowed if it was discontinued for reasons other than primary nonresponse more than 8 weeks [or five half-lives, whichever is longer] from first dose) and at least 12 weeks of nonbiologic, conventional synthetic disease-modifying antirheumatic drug (csDMARD) therapy prior to screening. Participants were required to remain on a stable dose of csDMARD therapy throughout the study.

Key exclusion criteria included the following: confirmed diagnosis of systemic lupus erythematosus, history of suicidal ideation or attempted suicide, active infection or significant history of infection, surgery on the joint chosen for biopsy or magnetic resonance imaging (MRI), history of other joint disease, and receipt of intraarticular corticosteroids, arthrocentesis, or synovial biopsy within 6 weeks of screening.

Full inclusion and exclusion criteria are included in Additional file 1 Item S1.

### Endpoints and assessments

The primary endpoint, safety, and tolerability of GSK2982772 60 mg b.i.d. or t.i.d. was assessed by monitoring AEs/SAEs, clinical laboratory tests, vital signs, and 12-lead electrocardiogram (ECG).

Blood samples were collected pre-dose on days 8 and 43, and 1, 2, 4, and 6 h post-dose on days 1, 8, and 43 to assess plasma concentrations of GSK2982772. Optional synovial biopsy samples were collected on days 1 and 43 using standard procedures for patients who consented to have biopsies collected [26]. Plasma and synovial biopsy samples were analyzed for GSK2982772 using high-performance liquid chromatography/tandem mass spectrometry (HPLC MS/MS).

Patient-reported outcomes included patient assessment of joint pain, which was determined from a numeric rating scale (0 [no pain] to 10 [most severe pain]). Fatigue was assessed on the Functional Assessment of Chronic Illness Therapy (FACIT) Fatigue Scale, a 13-point fatigue questionnaire [27].

Clinical disease assessments included DAS28-CRP [28] and ACR20/50/70 responses [29]. DAS28 is a composite score, the components of which are tender/painful joint count, swollen joint count, CRP level, and Patient’s Global Assessment of Disease. ACR20/50/70 response measures 20%, 50%, or 70% improvement in tender and swollen joint counts and in at least three of five core set measures: patient and physician global assessments of disease activity, patient-reported pain, disability (measured with the Health Assessment Questionnaire-Disability Index [HAQ-DI]), and an acute-phase reactant.

To evaluate structural joint inflammation and damage, gadolinium contrast-enhanced MRI of each patient’s most affected hand and wrist was performed pre-dose on days 1, 43, and 85. Synovitis, osteitis, and bone erosion were scored using the Outcome Measures in Rheumatology (OMERACT) Rheumatoid Arthritis MRI Scoring System (RAMRIS) [30], and cartilage loss was scored using the Cartilage Loss Scoring System (CARLOS) [31]. All MRI images were scored centrally by a single, expert radiologist blinded to visit order.

PD assessments in blood included mRNA expression and biomarkers indicative of RA disease activity such as the Vectra Disease Activity (DA) score and its components.

### Data and statistical analysis

This was a safety study and therefore not powered to test predefined differences in clinical efficacy or biomarkers.

The safety population consisted of all patients who received at least one dose of study treatment (safety, efficacy, and biomarker analyses). The PK populations for GSK2982772 consisted of patients in the safety population who received an active dose and for whom a GSK2982772 PK sample was obtained and analyzed.

The primary safety and tolerability analysis was based on review and display of adverse events (AEs), clinical laboratory values, vital signs, and ECG. For efficacy analyses, change from baseline data was analyzed using the mixed model repeated measures approach accounting for baseline, treatment, and visit. Biomarker endpoints (except Vectra DA) were transformed before analyzing, and their results are expressed as percentage changes. Frequencies and standard errors were reported by treatment group and visit for categorical endpoints. Additional details on statistical analyses performed are included in Additional file 1 Item S2.

## Results

### Disposition and demographics

**Fig. 1:**
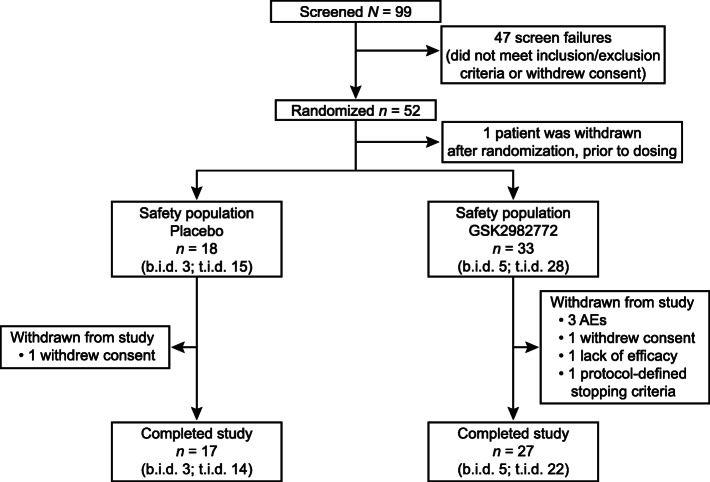
Patient disposition. AE, adverse event; b.i.d., twice daily; t.i.d., three times daily

A total of 52 patients were randomized (placebo, 18; GSK2982772, 34). All 18 participants in the placebo group (3 b.i.d., 15 t.i.d.) and 33 in the GSK2982772 group (5 b.i.d., 28 t.i.d.) received treatment and were included in the safety population (Fig. 1). A total of 44 participants completed the study, 17 in the placebo group (3 b.i.d., 14 t.i.d.) and 27 in the GSK2982772 group (5 b.i.d., 22 t.i.d.), and seven patients were withdrawn (Fig. 1). Treatment compliance, calculated from dispensed and returned tablet data, ranged from 95 to 100% for the placebo group, 96 to 100% for the GSK2982772 60 mg b.i.d. group, and 70 to 104% for the GSK2982772 60 mg t.i.d. group. Demographic characteristics, disease severity, and concurrent medical conditions showed no important differences between treatment groups (Table 1). A slight difference in mean baseline time since diagnosis was observed; however, median values were similar.

**Table 1:** Demographics and baseline characteristics (combined b.i.d. and t.i.d. dosing)

Demographics	Placebo (*n* = 18)	GSK2982772 (*n* = 33)
Age, years (mean [SD])^a^	53.1 (7.28)	54.8 (10.72)
Sex, *n* (%)		
Female	15 (83)	27 (82)
Male	3 (17)	6 (18)
BMI, kg/m^2^ (mean [SD])	26.5 (4.35)	27.8 (4.48)
Ethnicity, *n* (%)		
Hispanic or Latino	0	2 (6)
Not Hispanic or Latino	18 (100)	31 (94)
Race detail		
American Indian or Alaskan native	0	1 (3)
White—White/Caucasian/European Heritage	18 (100)	32 (97)
Time since formal diagnosis of RA, years		
Mean (SD)	4.5 (3.27)	7.3 (7.24)
Median (min, max)	3.9 (0.6, 12.9)	4.4 (0.4, 31.1)
Time since onset of first musculoskeletal symptoms, years		
Mean (SD)	6.6 (4.30)	9.5 (8.44)
Median (min, max)	5.5 (2.1, 14.4)	7.8 (0.6, 33.1)
Baseline medical conditions, *n* (%)		
Cardiovascular risk factors	8 (44)	12 (36)
Other risk factors	11 (61)	19 (58)
Baseline (day 1) DAS28-CRP		
Mean (SD)	5.4 (1.17)	5.2 (0.76)
Median (min, max)^a^	5.5 (1.9, 7.2)	5.1 (3.7, 7.1)
Baseline (day 1) CRP		
Mean (SD)	19.8 (15.52)	14.4 (19.47)
Median (min, max)^a^	15.1 (1.3, 53.5)	9.4 (0.9, 98.2)
Baseline (day 1) RAMRIS		
Synovitis		
Mean (SD)	6.4 (4.70)	10.5 (4.97)
Median (min, max)	6.0 (0, 15)	10.0 (2, 20)
Bone erosion		
Mean (SD)	11.5 (11.02)	16.5 (13.36)
Median (min, max)	7.0 (1, 40)	16.0 (1, 54)
Osteitis		
Mean (SD)	5.3 (7.97)	6.4 (9.61)
Median (min, max)	1.0 (0, 28)	2.0 (0, 36)
Baseline (day 1) CARLOS		
Mean (SD)	4.7 (9.32)	6.0 (9.06)
Median (min, max)	0 (0, 34)	1.5 (0, 36)
Baseline (day 1) Vectra DA		
Mean (SD)	50.8 (14.85)	50.9 (13.39)
Median (min, max)	51.5 (21, 69)	49.0 (29, 89)
Baseline (day 1) HAQ-DI		
Mean (SD)	1.3 (0.79)	1.2 (0.61)
Median (min, max)	1.6 (0.0, 2.3)	1.4 (0.0, 2.1)
Baseline (day 1) FACIT-Fatigue		
Mean (SD)	24.3 (9.92)	32.6 (10.18)
Median (min, max)	23.5 (8, 50)	32.5 (16, 52)

Data are expressed as mean (SD) unless otherwise stated

^a^Minimum values are outside the inclusion criteria because minimum values for inclusion were assessed at screening, and these data represent data collected at the baseline visit (day 1)

*b.i.d.*, twice daily; *BMI*, body mass index; *CARLOS*, Cartilage Loss Scoring System; *CRP*, C-reactive protein; *DAS28-CRP*, disease activity score for 28 joints using CRP value; *FACIT*, Functional Assessment of Chronic Illness Therapy; *HAQ-DI*, Health Assessment Questionnaire-Disability Index; *RA*, rheumatoid arthritis; *RAMRIS*, Rheumatoid Arthritis MRI Scoring System; *SD*, standard deviation; *t.i.d.*, three times daily; *Vectra DA*, Vectra disease activity

### Safety and tolerability

The frequency of AEs was similar across treatment groups, with AEs reported in 13 (72%) patients in the placebo group (100%, *n* = 3 for b.i.d.; 67%, *n* = 10 for t.i.d.), and 20 (61%) patients in the GSK2982772 group (60%, *n* = 3 for b.i.d., 61%, *n* = 17 for t.i.d.) (Table 2). The most commonly reported AEs occurring in > 10% of patients in either treatment group after combining the b.i.d. and t.i.d. regimens were arthralgia (11% in the combined placebo group and 6% in the combined GSK2982772 group), headache (11% in the combined placebo group and 3% in the combined GSK2982772 group), and peripheral swelling (11% in the combined placebo group and 0% in GSK2982772 group). No AEs in the combined GSK2982772 group occurred at a frequency > 10%.

**Table 2:** Adverse events (safety population)

	Placebo b.i.d. (*n* = 3)	Placebo t.i.d. (*n* = 15)	Placebo total (*n* = 18)	GSK2982772 60 mg b.i.d. (*n* = 5)	GSK2982772 60 mg t.i.d. (*n* = 28)	GSK2982772 60 mg total (*n* = 33)
Any AE, *n* (%)	3 (100)	10 (67)	13 (72)	3 (60)	17 (61)	20 (61)
Any SAE, *n* (%)	0	0	0	0	2 (7)	2 (6)
Eye disorder						
Retinal vein thrombosis	0	0	0	0	1 (4)	1 (3)
Injury, poisoning and procedural complications						
Multiple fractures	0	0	0	0	1 (4)	1 (3)
Any AE leading to withdrawal, *n* (%)	0	0	0	0	3 (11)	3 (9)
Infections and infestations						
Bronchitis	0	0	0	0	1 (4)	1 (3)
Eye disorder						
Retinal vein thrombosis (SAE)	0	0	0	0	1 (4)	1 (3)
Injury, poisoning, and procedural complications						
Multiple fractures (SAE)	0	0	0	0	1 (4)	1 (3)
Any treatment-related AEs, *n* (%)	3 (100)	2 (13)	5 (28)	1 (20)	6 (21)	7 (21)
Severe	0	0	0	0	1 (4)	1 (3)
Moderate	1 (33)	1 (7)	2 (11)	1 (20)	4 (14)	5 (15)
Mild	2 (67)	1 (7)	3 (17)	0	1 (4)	1 (3)
AEs occurring in > 10% of combined groups, *n* (%)						
Musculoskeletal and connective tissue disorders						
Arthralgia			2 (11)			2 (6)
Nervous system disorders						
Headache			2 (11)			1 (3)
General disorders and administration site conditions						
Peripheral swelling^a^			2 (11)			0

^a^Included swelling in the lower limbs and hands

*AE*, adverse event; *b.i.d.*, twice daily; *SAE*, serious adverse event; *t.i.d.*, three times daily

Two serious AEs were recorded in the GSK2982772 t.i.d. group (Table 2). One patient had multiple fractures (tibia, upper limb, and ulna) resulting from a fall and was withdrawn from treatment; the event was not considered related to study treatment. The other patient had an event of severe visual disturbance and retinal vein thrombosis at day 66 that was considered related to study treatment by the principal investigator. Confounders included the underlying indication of RA and elevated cholesterol. The patient was withdrawn from treatment and the study. Another significant AE observed with GSK2982772 t.i.d. was a case of moderate bronchitis that was considered related to study treatment and resulted in withdrawal from the study.

Most treatment-related AEs were mild or moderate in intensity (Table 2). All AEs and treatment-related AEs in both placebo groups and the GSK2982772 b.i.d. group were mild or moderate; treatment-related AEs classified as severe were observed in one patient (4%) in the GSK2982772 t.i.d. group (alopecia areata [onset at day 49, subject was not withdrawn nor study medication amended] and retinal vein thrombosis [at day 66, resulted in withdrawal from the study]).

Changes in hematology or clinical chemistry measurements, ECGs, and vital signs were as expected for the RA population and did not differ between placebo and treatment groups. One patient with a history of ventricular arrhythmia treated with GSK2982772 t.i.d. had a clinically significant abnormal AE of sinus arrhythmia in the final follow-up period (day 113). A low percentage of patients experienced elevated diastolic blood pressure (BP) (11% placebo, 9% GSK2982772) or systolic BP (6% placebo, 9% GSK2982772). No suicidality was reported during any part of the study.

### Clinical efficacy

**Fig. 2:**
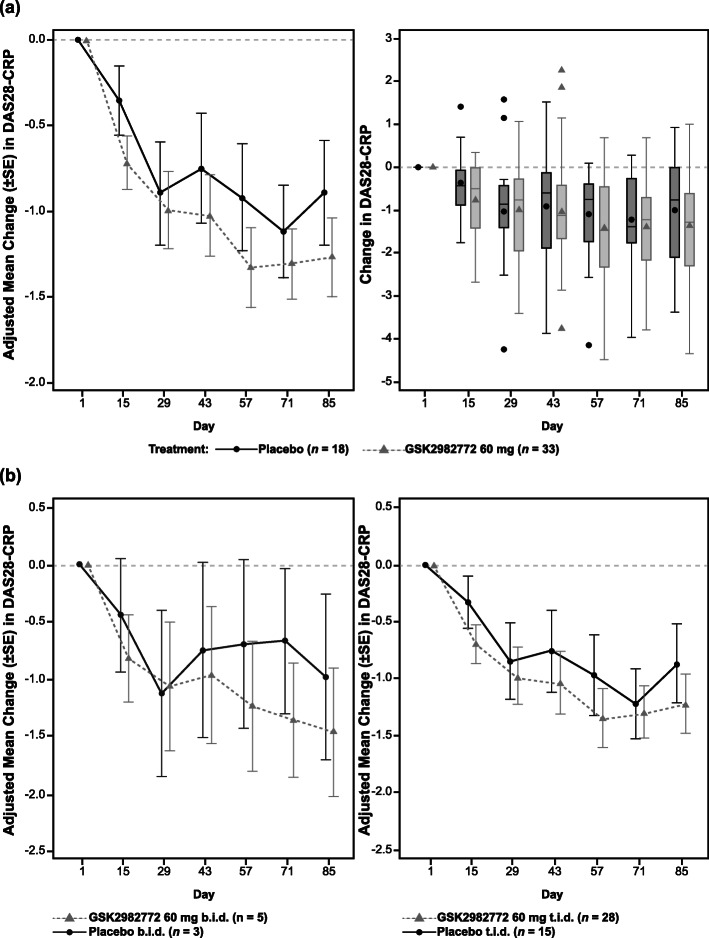
DAS28-CRP. **a** DAS28-CRP adjusted mean change from baseline and unadjusted box and whisker plot of unadjusted change from baseline by treatment over time (combined b.i.d. and t.i.d. dosing). **b** DAS28-CRP adjusted mean change from baseline by dosing regimen. b.i.d., twice daily; CRP, C-reactive protein; DAS28-CRP, disease activity score for 28 joints using CRP value; t.i.d., three times daily

**Fig. 3:**
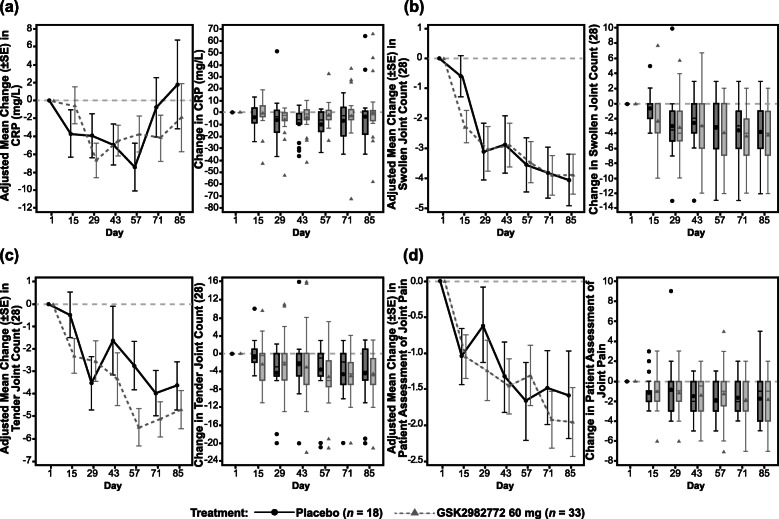
Clinical efficacy parameters (combined b.i.d. and t.i.d. dosing). Adjusted mean change from baseline and unadjusted box and whisker plot of unadjusted change from baseline by treatment over time. **a** C-reactive protein. **b** Swollen joint count (28). **c** Tender joint count (28). **d** Patient assessment of joint pain. b.i.d., twice daily; CRP, C-reactive protein; t.i.d., three times daily

**Fig. 4:**
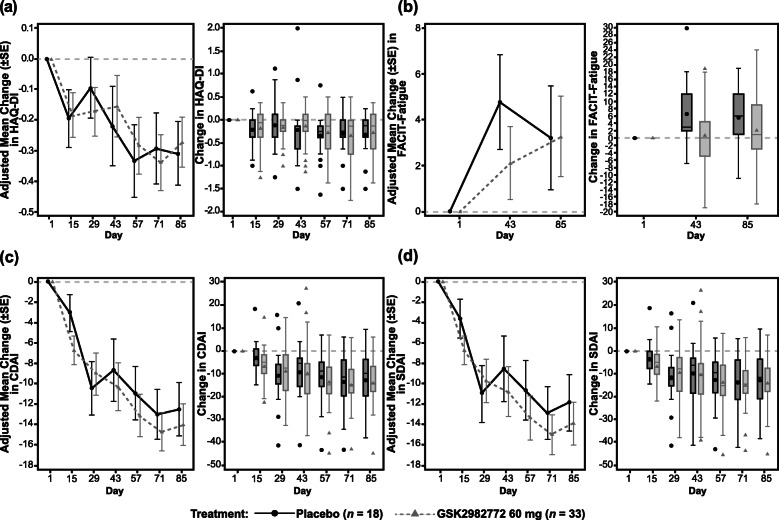
Health outcomes measures (combined b.i.d. and t.i.d. dosing). Adjusted mean change from baseline and unadjusted box and whisker plot of unadjusted change from baseline by treatment over time. **a** HAQ-DI. **b** FACIT fatigue. **c** Clinical disease activity index (CDAI). **d** Simple disease activity index (SDAI). b.i.d., twice daily; FACIT, Functional Assessment of Chronic Illness Therapy; HAQ-DI, Health Assessment Questionnaire-Disability Index; t.i.d., three times daily

Improvement in DAS28-CRP scores compared to baseline was observed with both GSK2982772 and placebo at the end of the treatment period (Table 3 and Fig. 2a, b). The difference in least squares (LS) mean change from baseline between GSK2982772 (combined data for the 60 mg b.i.d. and t.i.d. groups) and placebo was − 0.38 (95% confidence interval [CI], − 1.15, 0.40) at day 85. No statistically significant differences were observed between the GSK2982772 60 mg b.i.d. and t.i.d. dosing regimens, or with GSK2982772 treatment vs. placebo. Changes in DAS28-CRP scores appeared to be driven by changes in tender joint count, with no differences observed in CRP levels or swollen joint counts between the placebo group and combined treatment groups (Fig. 3a–c). Patient assessed joint pain (Fig. 3d) as well as composite health outcomes including change from baseline in the HAQ-DI [32] FACIT fatigue mean score, clinical disease activity index, and simple disease activity index were similar in the GSK2982772 group and the placebo group (Fig. 4a–d).

**Table 3:** Change from baseline in DAS28-CRP scores at day 85 (MMRM analysis)

Treatment regimen	*N* (*n*)	LS mean (SE)	95% CI for LS mean	Difference from placebo	95% CI for differences
Change from baseline at day 85					
Placebo (b.i.d. and t.i.d.)	18 (15)	−0.90 (0.31)	(−1.51, −0.28)		
GSK2982772 (b.i.d. and t.i.d.)	33 (27)	−1.27 (0.23)	(−1.73, −0.81)	−0.38	(−1.15, 0.40)
Change from baseline at day 85 by treatment regimen					
Placebo (b.i.d.)	3 (3)	−1.00 (0.72)	(−2.45, 0.46)		
GSK2982772 (b.i.d.)	5 (5)	−1.47 (0.56)	(−2.60, −0.35)	− 0.48	(−2.33, 1.37)
Placebo (t.i.d.)	15 (12)	−0.87 (0.35)	(−1.57, − 0.17)		
GSK2982772 (t.i.d.)	28 (22)	−1.23 (0.26)	(−1.75, −0.71)	−0.36	(− 1.23, 0.52)

*b.i.d*., twice daily; *CI*, confidence interval; *DAS28-CRP*, disease activity score for 28 joints using CRP value; *LS mean*, least squares mean; *MMRM*, mixed model repeated measures; *t.i.d*., three times daily

The proportion of patients achieving ACR20 (placebo 39%, GSK2982772 45%), ACR50 (placebo 17%, GSK2982772 15%), and ACR70 (placebo 11%, GSK2982772 12%) responses at day 85 were also similar between the two groups (Additional file 1 Figure S2).

### MRI

**Fig. 5:**
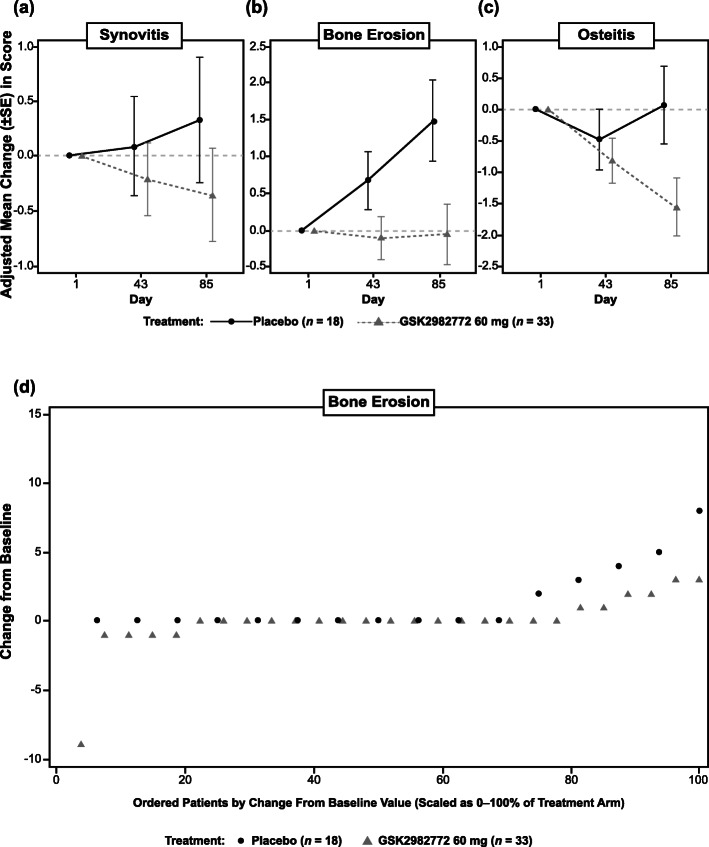
RAMRIS parameters. Adjusted mean change from baseline and unadjusted box and whisker plot of unadjusted change from baseline by treatment over time. **a** Synovitis. **b** Bone erosion. **c** Osteitis (combined b.i.d. and t.i.d. dosing). **d** Cumulative plot probability of change from baseline in bone erosion total score on day 85. b.i.d., twice daily; RAMRIS, Rheumatoid Arthritis MRI Scoring System; t.i.d., three times daily

RAMRIS synovitis improved slightly in the GSK2982772 treatment group and showed some deterioration in the placebo group (Fig. 5a). RAMRIS erosion scores deteriorated from baseline in the placebo group but not in the GSK2982772 group (Fig. 5b); the difference from placebo in LS mean score was − 1.5 (95% CI, − 2.9, − 0.1) at day 85. One patient in the GSK2982772 treatment group who showed a large improvement in erosions may have been a major contributor to the observed difference between the two groups (Fig. 5d). RAMRIS osteitis appeared to show improvement with GSK2982772 treatment (Fig. 5c).

A change in CARLOS score from baseline was observed in only one of 46 patients with postbaseline data. This patient (GSK2982772 60 mg t.i.d.) showed a two-point worsening of the score at both days 43 and 85.

### Pharmacokinetics of GSK2982772

Overall median pre-dose plasma concentrations were higher for the t.i.d. regimen than for the b.i.d. regimen (~ 1.7-fold, ~ 10-fold, and ~ 5.5-fold higher on days 9, 43, and 85, respectively).

Trough synovial tissue samples were only available for three patients, of whom two had quantifiable levels of GSK2982772 at day 43. The GSK2982772 pre-dose concentration in synovial tissue was of a similar order of magnitude to the corresponding patient’s pre-dose plasma concentration.

### Pharmacodynamics

#### Biomarkers

The blood biomarker mean Vectra DA score at day 1 was 50.8 and 50.9 for placebo and GSK2982772 (combined b.i.d. and t.i.d.) groups, respectively, indicating high disease activity at baseline. No clinically relevant changes from baseline were observed for either group at day 43 or day 85 or between the two GSK2982772 dosing groups (Additional file 1 Figure S3a). GSK2982772 treatment resulted in a small reduction over time for S100A8/S100A9 (Additional file 1 Figure S3b). No major changes were observed for other examined biomarkers (Additional file 1 Table S1). Because of the small number of synovial biopsy samples, biomarker data in synovial tissue were not available for comparison with blood data.

## Discussion

This was the first study of an RIPK1 inhibitor in patients with RA. Repeat doses of GSK2982772 60 mg b.i.d. or t.i.d. administration up to 85 days were well tolerated with no safety signals observed in patients with moderate to severe RA. No differences were noted in AEs between active treatment and placebo groups. Most AEs were mild or moderate, and no differences were noted between the b.i.d. and t.i.d. doses of GSK2982772. The study did not show any significant difference between GSK2982772 and placebo treatment for changes in disease activity, radiological progression, or inflammatory markers.

In the current study, the b.i.d. and t.i.d. regimens of GSK2982772 were similar with respect to safety, efficacy, and PD assessments. However, the study was not powered to investigate differences in DAS28-CRP responses between the two doses, and the numbers in the b.i.d. cohort were too small for any strong conclusions to be drawn. Higher GSK2982772 exposure levels were achieved with 60 mg t.i.d. dosing than with b.i.d. dosing. Although drug levels were detectable in two of only three available synovial tissue samples, concentrations of GSK2982772 in synovial tissue appeared to be of a similar order of magnitude to the concentrations in plasma indicating that GSK2982772 can penetrate into the effect site compartment.

Changes in disease activity from baseline, as measured by DAS28-CRP and ACR20/50/70 responses, improved with GSK2982772, but did not differ significantly compared to placebo. The placebo effect observed in our study was in line with that noted in other studies in patients with moderate to severe RA [33–43]. However, in contrast to our study, other small molecule studies (i.e., tofacitinib, baricitinib, upadacitinib, filgotinib, fenebrutinib) showed a significant improvement in disease activity vs. placebo [33–43].

GSK2982772 treatment resulted in a small improvement in the RAMRIS synovitis score compared to placebo; however, the difference in means may have been largely influenced by two patients, with most patients showing a similar response across treatment groups. Bone erosion worsened in the placebo group over the study duration, with little change in the GSK2982772 group, but one patient in the GSK29822772 group, who showed a large improvement, may have contributed to much of the mean difference between treatment groups. Alternatively, this finding may support other studies that have suggested a potential role for RIPK1 inhibition in the suppression of osteoclastogenesis [22, 44].

A mean Vectra DA score of ~ 51 at baseline in both treatment groups was indicative of the high overall disease activity of the recruited patients at baseline. The overall Vectra DA score remained unchanged in both groups over time. Minor improvements in some biomarkers were observed in the active treatment group, but these changes did not translate into a greater clinical benefit.

In conclusion, this study suggested that administration of GSK2982772 60 mg b.i.d. or t.i.d. for 84 days in patients with moderate to severe RA was safe and well tolerated. However, inhibition of RIPK1 activity with GSK2982772 did not lead to differences in measures of efficacy and joint damage compared with placebo.

## Supplementary Information

**Additional file 1: Supplementary Figure S1.** Chemical structure of GSK2982772. **Supplementary Item-S1.** Full inclusion and exclusion criteria. **Supplementary Item-S2.** Statistical analysis. **Supplementary Figure S2.** Proportion (±SE) of patients achieving ACR 20/50/70 (combined b.i.d. and t.i.d. dosing). **Supplementary Figure S3.** Biomarkers (combined b.i.d. and t.i.d. dosing). **Supplementary Table-S1.** Summary of inflammatory biomarkers in blood.

## Data Availability

Within 6 months of this publication, the dataset(s) supporting the conclusions of this article will be available in the Clinical Study Data Request repository, proposals should be submitted to www.clinicalstudydatarequest.com. A data access agreement will be required.

## Abbreviations

AE: Adverse event
BMI: Body mass index
C3L1: Chitinase 3 like-1
CARLOS: Cartilage Loss Scoring System
CI: Confidence interval
CRP: C-reactive protein
csDMARD: Conventional synthetic disease-modifying antirheumatic drug
DAS: Disease activity score
DAS28-CRP: Disease activity score for 28 joints using CRP value
DRC: Data review committee
ECG: Electrocardiogram
EGF: Epidermal growth factor
EULAR: European League Against Rheumatism
FACIT: Functional Assessment of Chronic Illness Therapy
GCSP: Global Clinical Safety and Pharmacovigilance
HPLC MS/MS: High-performance liquid chromatography/tandem mass spectrometry
LS: Least squares
MCP1: Monocyte chemotactic protein
MMIF: Macrophage migration inhibitory factor
MMP: Matrix metalloproteinase
MMRM: Mixed model repeated measures
MRI: Magnetic resonance imaging
NIHR: National Institute for Health Research
OMERACT: Outcome Measures in Rheumatology
PD: Pharmacodynamics
PK: Pharmacokinetics
RA: Rheumatoid arthritis
RAMRIS: Rheumatoid Arthritis MRI Scoring System
RIPK1: Receptor-interacting protein kinase 1
SAE: Serious adverse event
SD: Standard deviation
SRT: Safety review team
TIMP1: Tissue inhibitor of metalloproteinase 1
TNF: Tumor necrosis factor
TNFR1: Tumor necrosis factor receptor type 1
VCAM1: Vascular cell adhesion molecule 1
VEGF: Vascular endothelial growth factor

## References

[1] Tak PP, Bresnihan B (2000). The pathogenesis and prevention of joint damage in rheumatoid arthritis: advances from synovial biopsy and tissue analysis. Arthritis Rheum.

[2] Emery P (2012). Optimizing outcomes in patients with rheumatoid arthritis and an inadequate response to anti-TNF treatment. Rheumatology (Oxford).

[3] Wijbrandts CA, Tak PP (2017). Prediction of response to targeted treatment in rheumatoid arthritis. Mayo Clin Proc.

[4] Smolen JS, Aletaha D, McInnes IB (2016). Rheumatoid arthritis. Lancet..

[5] Choy EH, Panayi GS (2001). Cytokine pathways and joint inflammation in rheumatoid arthritis. N Engl J Med.

[6] Tracey D, Klareskog L, Sasso EH, Salfeld JG, Tak PP (2008). Tumor necrosis factor antagonist mechanisms of action: a comprehensive review. Pharmacol Ther.

[7] Palombella VJ, Conner EM, Fuseler JW, Destree A, Davis JM, Laroux FS (1998). Role of the proteasome and NF-kappaB in streptococcal cell wall-induced polyarthritis. Proc Natl Acad Sci U S A.

[8] Miagkov AV, Kovalenko DV, Brown CE, Didsbury JR, Cogswell JP, Stimpson SA (1998). NF-kappaB activation provides the potential link between inflammation and hyperplasia in the arthritic joint. Proc Natl Acad Sci U S A.

[9] Matmati M, Jacques P, Maelfait J, Verheugen E, Kool M, Sze M (2011). A20 (TNFAIP3) deficiency in myeloid cells triggers erosive polyarthritis resembling rheumatoid arthritis. Nat Genet.

[10] Ofengeim D, Yuan J (2013). Regulation of RIP1 kinase signalling at the crossroads of inflammation and cell death. Nat Rev Mol Cell Biol.

[11] Humphries F, Yang S, Wang B, Moynagh PN (2015). RIP kinases: key decision makers in cell death and innate immunity. Cell Death Differ.

[12] Zhou W, Yuan J (2014). Necroptosis in health and diseases. Semin Cell Dev Biol.

[13] Berger SB, Harris P, Nagilla R, Kasparcova V, Hoffman S, Swift B (2015). Characterization of GSK'963: a structurally distinct, potent and selective inhibitor of RIP1 kinase. Cell Death Discov..

[14] Berger SB, Kasparcova V, Hoffman S, Swift B, Dare L, Schaeffer M (2014). Cutting edge: RIP1 kinase activity is dispensable for normal development but is a key regulator of inflammation in SHARPIN-deficient mice. J Immunol.

[15] Boisson B, Laplantine E, Dobbs K, Cobat A, Tarantino N, Hazen M (2015). Human HOIP and LUBAC deficiency underlies autoinflammation, immunodeficiency, amylopectinosis, and lymphangiectasia. J Exp Med.

[16] O’Donnell MA, Hase H, Legarda D, Ting AT (2012). NEMO inhibits programmed necrosis in an NFκB-independent manner by restraining RIP1. PLoS One.

[17] Gerlach B, Cordier SM, Schmukle AC, Emmerich CH, Rieser E, Haas TL (2011). Linear ubiquitination prevents inflammation and regulates immune signalling. Nature..

[18] Raghav SK, Gupta B, Agrawal C, Chaturvedi VP, Das HR. Expression of TNF-alpha and related signaling molecules in the peripheral blood mononuclear cells of rheumatoid arthritis patients. Mediators Inflamm. 2006;2006:12682. 10.1155/MI/2006/12682 PMC1592599 16951485

[19] Hou J, Ju J, Zhang Z, Zhao C, Li Z, Zheng J (2019). Discovery of potent necroptosis inhibitors targeting RIPK1 kinase activity for the treatment of inflammatory disorder and cancer metastasis. Cell Death Discov.

[20] Roderick JE, Hermance N, Zelic M, Simmons MJ, Polykratis A, Pasparakis M (2014). Hematopoietic RIPK1 deficiency results in bone marrow failure caused by apoptosis and RIPK3-mediated necroptosis. Proc Natl Acad Sci U S A.

[21] Wagner PN, Shi Q, Salisbury-Ruf CT, Zou J, Savona MR, Fedoriw Y (2019). Increased Ripk1-mediated bone marrow necroptosis leads to myelodysplasia and bone marrow failure in mice. Blood..

[22] Jhun J, Lee SH, Kim SY, Ryu J, Kwon JY, Na HS (2019). RIPK1 inhibition attenuates experimental autoimmune arthritis via suppression of osteoclastogenesis. J Transl Med.

[23] Harris PA, Berger SB, Jeong JU, Nagilla R, Bandyopadhyay D, Campobasso N (2017). Discovery of a first-in-class receptor interacting protein 1 (RIP1) kinase specific clinical candidate (GSK2982772) for the treatment of inflammatory diseases. J Med Chem.

[24] Weisel K, Scott NE, Tompson DJ, Votta BJ, Madhavan S, Povey K (2017). Randomized clinical study of safety, pharmacokinetics, and pharmacodynamics of RIPK1 inhibitor GSK2982772 in healthy volunteers. Pharmacol Res Perspect.

[25] Aletaha D, Neogi T, Silman AJ, Funovits J, Felson DT, Bingham CO (2010). 2010 rheumatoid arthritis classification criteria: an American College of Rheumatology/European League Against Rheumatism collaborative initiative. Arthritis Rheum.

[26] van de Sande MGH, Gerlag DM, Lodde BM, van Baarsen LGM, Alivernini S, Codullo V (2011). Evaluating antirheumatic treatments using synovial biopsy: a recommendation for standardisation to be used in clinical trials. Ann Rheum Dis.

[27] Webster K, Cella D, Yost K (2003). The Functional Assessment of Chronic Illness Therapy (FACIT) measurement system: properties, applications, and interpretation. Health Qual Life Outcomes.

[28] van Riel PL (2014). The development of the disease activity score (DAS) and the disease activity score using 28 joint counts (DAS28). Clin Exp Rheumatol.

[29] American College of Rheumatology Committee to Reevaluate Improvement Criteria (2007). A proposed revision to the ACR20: the hybrid measure of American College of Rheumatology response. Arthritis Rheum.

[30] Østergaard M, Peterfy CG, Bird P, Gandjbakhch F, Glinatsi D, Eshed I (2017). The OMERACT rheumatoid arthritis magnetic resonance imaging (MRI) scoring system: updated recommendations by the OMERACT MRI in Arthritis Working Group. J Rheumatol.

[31] Peterfy CG, DiCarlo JC, Olech E, Bagnard MA, Gabriele A, Gaylis N (2012). Evaluating joint-space narrowing and cartilage loss in rheumatoid arthritis by using MRI. Arthritis Res Ther.

[32] Bruce B, Fries JF (2003). The Stanford Health Assessment Questionnaire: a review of its history, issues, progress, and documentation. J Rheumatol.

[33] Cohen S, Tuckwell K, Katsumoto TR, Zhao R, Lee C, Berman A, et al. Fenebrutinib compared to placebo and adalimumb in patients with inadequate response to either methotrexate therapy or prior TNF therapy: phase 2 study. American College of Rheumatology annual meeting; November 8–13, 2019; Atlanta, GA. Abstract OP0025.

[34] Combe B, Kivitz A, Tanaka Y, van der Heijde D, Matzkies F, Bartok B, et al. Efficacy and safety of filgotinib for patients with rheumatoid arthritis with inadequate response to methotrexate: FINCH1 primary outcome results. American College of Rheumatology annual meeting; November 8–13, 2019; Atlanta, GA. Abstract LB0001.

[35] Dougados M, van der Heijde D, Chen YC, Greenwald M, Drescher E, Liu J (2017). Baricitinib in patients with inadequate response or intolerance to conventional synthetic DMARDs: results from the RA-BUILD study. Ann Rheum Dis.

[36] Fleischmann R, Kremer J, Cush J, Schulze-Koops H, Connell CA, Bradley JD (2012). Placebo-controlled trial of tofacitinib monotherapy in rheumatoid arthritis. N Engl J Med.

[37] Fleischmann R, Pangan AL, Song IH, Mysler E, Bessette L, Peterfy C (2019). Upadacitinib versus placebo or adalimumab in patients with rheumatoid arthritis and an inadequate response to methotrexate: results of a phase III, double-blind, randomized controlled trial. Arthritis Rheum.

[38] Kremer J, Li ZG, Hall S, Fleischmann R, Genovese M, Martin-Mola E (2013). Tofacitinib in combination with nonbiologic disease-modifying antirheumatic drugs in patients with active rheumatoid arthritis: a randomized trial. Ann Intern Med.

[39] Kremer JM, Cohen S, Wilkinson BE, Connell CA, French JL, Gomez-Reino J (2012). A phase IIb dose-ranging study of the oral JAK inhibitor tofacitinib (CP-690,550) versus placebo in combination with background methotrexate in patients with active rheumatoid arthritis and an inadequate response to methotrexate alone. Arthritis Rheum.

[40] Smolen JS, Pangan AL, Emery P, Rigby W, Tanaka Y, Vargas JI (2019). Upadacitinib as monotherapy in patients with active rheumatoid arthritis and inadequate response to methotrexate (SELECT-MONOTHERAPY): a randomised, placebo-controlled, double-blind phase 3 study. Lancet..

[41] Taylor PC, Keystone EC, van der Heijde D, Weinblatt ME, Del Carmen ML, Gonzaga JR (2017). Baricitinib versus placebo or adalimumab in rheumatoid arthritis. N Engl J Med.

[42] van der Heijde D, Tanaka Y, Fleischmann R, Keystone E, Kremer J, Zerbini C (2013). Tofacitinib (CP-690,550) in patients with rheumatoid arthritis receiving methotrexate: twelve-month data from a twenty-four-month phase III randomized radiographic study. Arthritis Rheum.

[43] van Vollenhoven RF, Fleischmann R, Cohen S, Lee EB, García Meijide JA, Wagner A (2012). Tofacitinib or adalimumab versus placebo in rheumatoid arthritis. N Engl J Med.

[44] Lamothe B, Lai Y, Xie M, Schneider MD, Darnay BG (2013). TAK1 is essential for osteoclast differentiation and is an important modulator of cell death by apoptosis and necroptosis. Mol Cell Biol.

