# Supplementary material for: A randomized, placebo-controlled experimental medicine study of RIPK1 inhibitor GSK2982772 in patients with moderate to severe rheumatoid arthritis
Source: Arthritis Res Ther. 2021 Mar 16;23:85. doi: 10.1186/s13075-021-02468-0 (PMC7962407; doi:10.1186/s13075-021-02468-0)
Supplement: Supplementary file 1 — Additional file 1: Supplementary Figure S1. Chemical structure of GSK2982772. Supplementary Item-S1. Full inclusion and exclusion criteria. Supplementary Item-S2. Statistical analysis. Supplementary Figure S2. Proportion (±SE) of patients achieving ACR 20/50/70 (combined b.i.d. and t.i.d. dosing). Supplementary Figure S3. Biomarkers (combined b.i.d. and t.i.d. dosing). Supplementary Table-S1. Summary of inflammatory biomarkers in blood. [file 13075_2021_2468_MOESM1_ESM.docx]

**Additional file 1**

**Supplementary Figure S1.** Chemical structure of GSK2982772


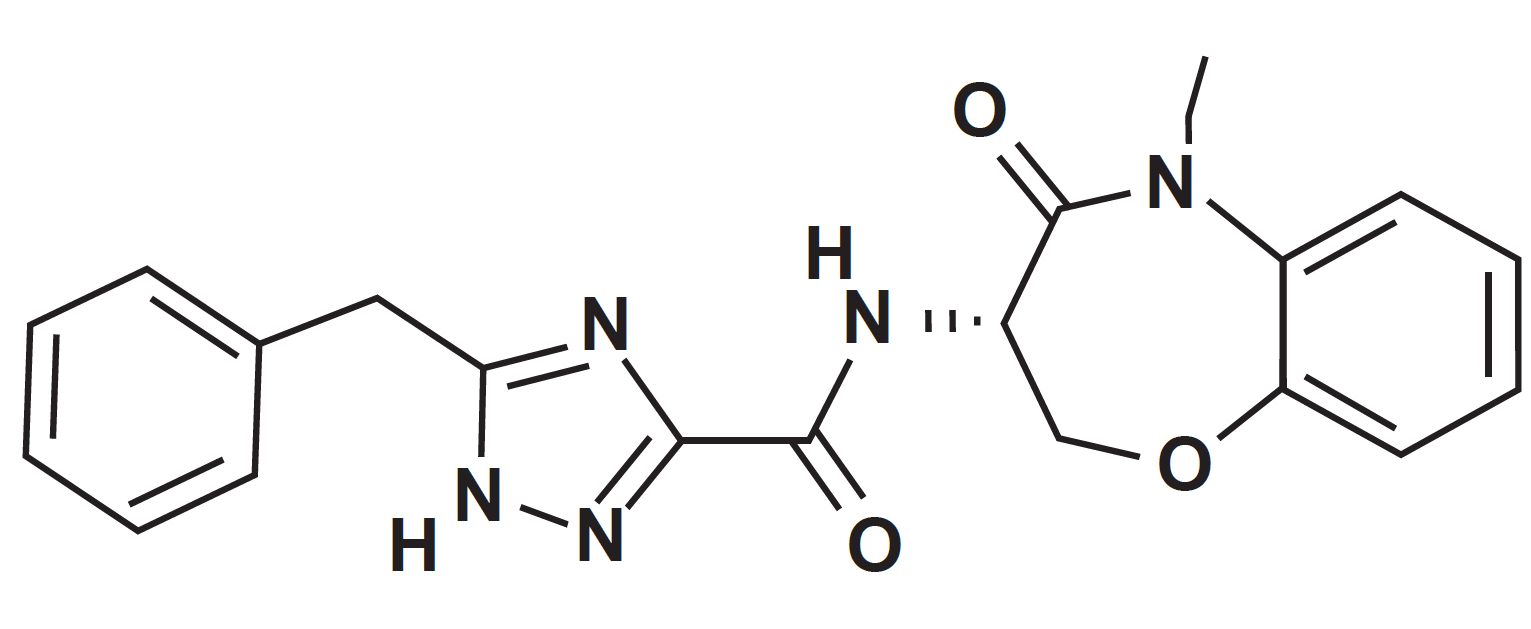


**Supplementary Item S1**

Full inclusion and exclusion criteria

Inclusion Criteria

**AGE**

1. Between 18 and 75 years of age inclusive, at the time of signing the informed consent.

**TYPE OF SUBJECT AND DIAGNOSIS INCLUDING DISEASE SEVERITY**

1. Subjects that do not have any medical conditions, other than moderate to severe RA that in the opinion of the Investigator put the subject at unacceptable risk or interfere with study assessments or integrity of the data. These medical conditions should be stable at the time of screening and are expected to remain stable for the duration of the study.
2. Subject has had a confirmed diagnosis of rheumatoid arthritis according to the revised 2010 American College of Rheumatology/European League Against Rheumatism ACR-EULAR classification criteria.
3. Disease duration of ≥12 weeks (time from onset of patient-reported symptoms of either pain or stiffness or swelling in hands, feet or wrists) at screening.
4. Swollen joint count of ≥4 (28-joint count) and tender joint count ≥4 (28-joint count) at screening.
5. Subject has a DAS28 CRP disease activity score of ≥3.2 and CRP ≥5.0 mg/L (≥4.76 nmol/L) at screening.
6. Subject must have received at least 12 weeks of non-biologic DMARD monotherapy or methotrexate (MTX)/DMARD combination therapy prior to screening AND must be on stable dose throughout the study.
7. Subject is naive to any biological therapies for RA

**OR**

Subject may have had previous exposure to a single anti-TNF biologic agent which was discontinued for reasons other than primary non-response more than 8 weeks (or 5 half-lives whichever is longer) from first dose. **Note: Exposure to a single anti-TNF is not required in addition to Inclusion #7 above.**

1. For subjects who have consented to synovial joint biopsy:
   1. Subject has an involved knee, wrist, or ankle suitable for biopsy, as assessed by a rheumatologist at screening.

**WEIGHT**

1. A body mass index (BMI) within range of 18.5 - 35 kg/m2 (inclusive) at screening

**SEX**

1. Male and female subjects

**Males:**

Male subjects with female partners of child-bearing potential must comply with contraception requirements.

**Females**:

A female subject is eligible to participate if she is not pregnant (as confirmed by a negative serum human chorionic gonadotrophin [hCG] test), not lactating, and at least one of the following conditions applies:

- 1. Non-reproductive potential defined as:
- Pre-menopausal females with one of the following:
  - - - Documented tubal ligation
- Documented hysteroscopic tubal occlusion procedure with follow-up confirmation of bilateral tubal occlusion
- Hysterectomy
- Documented Bilateral Oophorectomy
- Postmenopausal defined as 12 months of spontaneous amenorrhea in questionable cases a blood sample with simultaneous follicle stimulating hormone (FSH) and estradiol levels consistent with menopause (refer to laboratory reference ranges for confirmatory levels). Females on hormone replacement therapy (HRT) and whose menopausal status is in doubt will be required to use one of the highly effective contraception methods if they wish to continue their HRT during the study. Otherwise, they must discontinue HRT to allow confirmation of post-menopausal status prior to study enrolment.

b. Reproductive potential and agrees to follow one of the options listed in the Modified List of Highly Effective Methods for Avoiding Pregnancy in Females of Reproductive Potential (FRP) from 30 days prior to the first dose of study medication and until at least 30 days after the last dose of study medication and completion of the follow-up visit.

The Investigator is responsible for ensuring that subjects understand how to properly use these methods of contraception.

**INFORMED CONSENT**

1. Capable of giving signed informed consent which includes compliance with the requirements and restrictions listed in the consent form and in this protocol.

Exclusion Criteria

**CONCURRENT CONDITIONS/MEDICAL HISTORY (INCLUDES LIVER FUNCTION AND QTc INTERVAL)**

1. Subject with a positive anti-double stranded deoxyribonucleic acid (DNA [anti-dsDNA]) and confirmed diagnosis of systemic lupus erythematosus (SLE).
2. Subject with current history of Suicidal Ideation Behaviour (SIB) as measured using the Columbia Suicide Severity Rating Scale (C-SSRS) or a history of attempted suicide.
3. An active infection, or a history of infections as follows:
   - Hospitalisation for treatment of infection within 60 days before first dose (Day 1).

Currently on any suppressive therapy for a chronic infection (such as pneumocystis, cytomegalovirus, herpes simplex virus, herpes zoster and atypical mycobacteria).

- Use of parenteral (IV or intramuscular) antibiotics (antibacterials, antivirals, antifungals, or antiparasitic agents) for an infection within 60 days before first dose.
- A history of opportunistic infections within 1 year of screening (e.g. pneumocystis jirovecii, CMV pneumonitis, aspergillosis). This does not include infections that may occur in immunocompetent individuals, such as fungal nail infections or vaginal candidiasis, unless it is of an unusual severity or recurrent nature.
- Recurrent or chronic infection or other active infection that, in the opinion of the Investigator might cause this study to be detrimental to the patient.
- History of TB, irrespective of treatment status.
- A positive diagnostic TB test at screening defined as a positive

QuantiFERON-TB Gold test or T-spot test. In cases where the QuantiFERON or T-spot test is indeterminate, the subject may have the test repeated once, but they will not be eligible for the study unless the second test is negative. In cases where the QuantiFERON or T-spot test is positive, but a locally-read follow up chest x-ray, shows no evidence of current or previous pulmonary tuberculosis, the subject may be eligible for the study at the discretion of the Investigator and GSK Medical Monitor.

1. QTc >450msec or QTc >480msec for subjects with bundle branch block at screening. The QTc is the QT interval corrected for heart rate according to either Bazett’s formula (QTcB), Fridericia’s formula (QTcF), or another method, machine or manual over read.

The specific formula that will be used to determine eligibility and discontinuation for an individual subject should be determined and documented prior to initiation of the study. In other words, several different formulae cannot be used to calculate the QTc for an individual subject and then the lowest QTc value used to include or discontinue the subject from the trial. For purposes of data analysis, QTcB, QTcF, another QT correction formula, or a composite of available values of QTc will be used as specified in the Reporting and Analysis Plan (RAP).

1. ALT >2xULN and bilirubin >1.5xULN (isolated bilirubin >1.5xULN is acceptable if bilirubin is fractionated and direct bilirubin <35%) at screening.
2. Current active or chronic history of liver or biliary disease (with the exception of Gilbert’s syndrome or asymptomatic gallstones).
3. Current or history of renal disease or estimated glomerular filtration rate (GFR) by Chronic Kidney Disease Epidemiology Collaboration equation (CKD-EPI) calculation <60 mL/min/1.73m^2^ at screening.
4. Hereditary or acquired immunodeficiency disorder, including immunoglobulin deficiency.
5. A major organ transplant (e.g., heart, lung, kidney, liver) or hematopoietic stem cell/marrow transplant.
6. Any planned surgical procedures including surgical joint procedures (e.g., intra- articular, tendon sheath, or bursal corticosteroid injections) during the study.
7. A history of malignant neoplasm within the last 5 years, except for adequately treated non-metastatic cancers of the skin (basal or squamous cell) or carcinoma in situ of the uterine cervix that has been fully treated and shows no evidence of recurrence.
8. Has undergone surgery including synovectomy or arthroplasty on the joint chosen for biopsy and/or magnetic resonance imaging (MRI).
9. The subject has a history of any other joint disease other than RA at the knee, wrist or ankle joint chosen for biopsy and/or MRI (e.g., gout, pseudogout, osteoarthritis).
10. Has undergone intra-articular corticosteroid injection, arthrocentesis or synovial biopsy on any joint within 6 weeks of screening.
11. A known allergy to lidocaine or other local anaesthetics (**Note**: only applies to subjects who consent for synovial biopsy procedures).
12. Contraindication to MRI scanning (as assessed by local MRI safety questionnaire) which includes but are not limited to:
    - Intracranial aneurysm clips (except Sugita) or other metallic objects,
    - History of intra-orbital metal fragments that have not been removed by a medical professional.
    - Pacemakers or other implanted cardiac rhythm management devices and non- MR compatible heart valves,
    - Inner ear implants,
    - History of claustrophobia which may impact participation.

**CONCOMITANT MEDICATIONS**

1. The subject has received treatment with the prohibited medications (see table below), or changes to those treatments, within the prescribed timeframe. If in doubt, or the therapy is not listed please consult with the medical monitor.
   - Other medications (including vitamins, herbal and dietary supplements) will be considered on a case-by-case basis, and will be allowed if in the opinion of the Investigator the medication will not interfere with the study procedures or compromise subject safety.

| **Prohibited Medications** | |
| --- | --- |
| **Therapy** | **Time period** |
| A change in dose of methotrexate or other DMARD | 12 weeks prior to screening until after the follow up visit (Day 112) |
| Greater than 10mg/day oral prednisolone (or  equivalent glucocorticoid) or a change in dose of corticosteroid | 4 weeks prior to screening until after the follow up visit (Day 112) |
| Intramuscular glucocorticoids (e.g.,  methylprednisolone ≤120 mg/month) | 4 weeks prior to screening until after the  follow up visit (Day 112) |
| Intra-articular corticosteroid injections | 6 weeks prior to screening and until after the follow up visit (Day 112) |
| Janus Kinase (JAK) Inhibitors | 4 weeks prior to screening until after the follow up visit (Day 112) |
| P-glycoprotein (Pgp) inhibitors including but not limited to amiodarone, azithromycin, captopril, carvedilol, clarithromycin, conivaptan, cyclosporine, diltiazem, dronedarone, erythromycin, felodipine, itraconazole, ketoconazole, lopinavir, ritonavir,  quercetin, quinidine, ranolazine, ticagrelor, verapamil [FDA, 2012] | 4 weeks prior to first dose (Day 1) until after the follow up visit (Day 112) |
| Narrow therapeutic index (NTI) CYP3A4 substrates including but not limited to alfentanil, astemizole, cisapride, cyclosporine, dihydroergotamine, ergotamine, fentanyl, pimozide, quinidine, sirolimus,  tacrolimus, terfenadine [FDA, 2012] | 4 weeks prior to the first dose (Day 1) until after the follow up visit (Day 112) |
| Biologic therapies for the treatment of rheumatoid arthritis not limited to anti-TNF biologics or other biologics, rituximab, anakinra, abatacept or  tocilizumab | At any time |
| Exposure to more than one anti-TNF biologic therapies for the treatment of RA including but not limited to anti-TNF biologics, infliximab, adalimumab, etanercept, certolizumab and golimumab  **Exception**: Exposure to a single anti-TNF-biologic for which the subject discontinued for a reason other than primary non-response is permitted | Cannot have been exposed to more than one anti-TNF biologic or be on at any time during the study  In the case of a single anti-TNF biologic for which the subject discontinued for a reason other than primary non-response, the subject must not be on for 8 weeks or 5 half lives (whichever is longer) prior to  first dose until after the follow up visit (Day 112) |
| Live vaccination | Live or attenuated vaccinations are not permitted within 30 days of randomization or plan to receive a vaccination during the study until follow-up visit. If indicated, non-live vaccines  (e.g., inactivated influenza vaccines) may be administered whilst receiving GSK2982772 based on an assessment of the benefit:risk (e.g., risk of decreased responsiveness). Investigators are expected to follow local and/or national guidelines with respect to vaccinations, including against pneumococcus and influenza, in subjects with RA. |

DMARD, disease-modifying antirheumatic drug; FDA, Food and Drug Administration; NTI, Narrow therapeutic index; RA, rheumatoid arthritis; TNF, Tumor necrosis factor:

**RELEVANT HABITS**

1. History of alcohol or drug abuse that would interfere with the ability to comply with the study.

**CONTRAINDICATIONS**

1. History of sensitivity to any of the study treatments, or components thereof or a history of drug or other allergy that, in the opinion of the Investigator or Medical Monitor, contraindicates their participation.
2. Received a live or attenuated vaccine within 30 days of randomization OR plan to receive a vaccination during the study until 5 half-lives (or 2 days) plus 30 days after receiving GSK2982772.
3. Contraindication to gadolinium contrast agent in accordance with local guidelines.
4. The subject has participated in a clinical trial and has received an investigational product within 30 days or 5 half-lives, whichever is longer before the first dose of study medication, or plans to take part in another clinical trial at the same time as participating in this clinical trial.

**DIAGNOSTIC ASSESSMENTS AND OTHER CRITERIA**

1. Haemoglobin <9 g/dL; haematocrit <30%, white blood cell count ≤3,000/mm3 (≤3.0 x 109/L); platelet count ≤100,000/µL (≤100 x 109/L); absolute neutrophil count ≤1.5 x 109/L at screening.
2. Presence of hepatitis B surface antigen (HBsAg), positive hepatitis C antibody test result at screening or within 3 months prior to first dose of study treatment. As potential for and magnitude of immunosuppression with this compound is unknown, subjects with presence of hepatitis B core antibody (HBcAb) should be excluded.
3. A positive serology for human immunodeficiency virus (HIV) 1 or 2 at screening.
4. Where participation in the study would result in donation of blood or blood products in excess of 500 mL within 3 months.
5. Exposure to more than 4 investigational medicinal products within 12 months prior to the first dose.

**Supplementary Item S2**

Statistical analysis

This was a safety study and therefore not powered to detect predefined differences in clinical efficacy or biomarkers. The primary objective of the study was safety and tolerability, and randomizing this number of patients gave adequate precision around observed safety event incidence rates. Using a Bayesian approach to determine the confidence interval (CI) around an observed safety event, we would assume a flat beta (1, 1) prior, and if we were to observe one safety event in 16, then the posterior distribution would be beta (2, 16). Thus, we could be 95% certain that the true probability of the safety event lay between 2% and 25%.

The safety population consisted of all patients who received at least one dose of study treatment (safety, efficacy, and biomarker analyses). The pharmacokinetic (PK) populations for GSK2982772 consisted of patients in the safety population who received an active dose and for whom a GSK2982772 PK sample was obtained and analyzed. The per-protocol population (Disease Activity Score in 28 Joints–C-reactive protein [DAS28-CRP] analysis) consisted of patients in the safety population after exclusion of patients with a protocol deviation that could have influenced the analysis.

The primary safety and tolerability analysis was based on review and display of adverse events (AEs), clinical laboratory values, vital signs, and electrocardiogram (ECG). All safety evaluations were based on the safety population.

For efficacy analyses, change from baseline of continuous endpoints was analyzed using mixed model repeated measures (MMRM) analysis with baseline, treatment, and their interactions with visit included as fixed effects and an unstructured covariance matrix. Biomarker endpoints were logged transformed before conducting the MMRM analysis, and their results expressed as percentage changes (with the exception of Vectra DA score). Frequencies and standard errors were reported by treatment group and visit for categorical endpoints (standard errors were calculated assuming a normal approximation).

**Supplementary Figure S2.** Proportion (±SE) of patients achieving ACR 20/50/70 (combined b.i.d. and t.i.d. dosing)


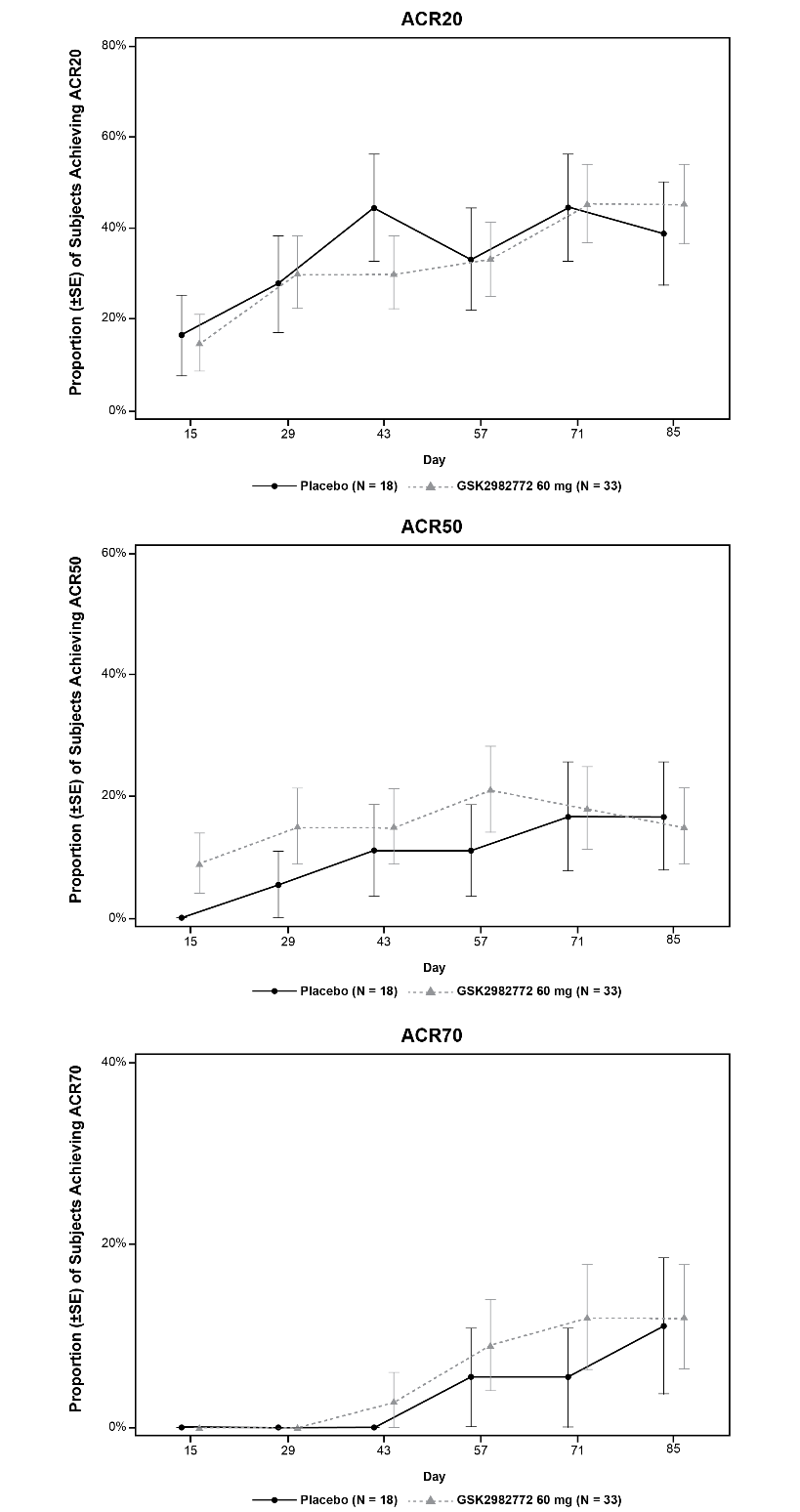


ACR, American College of Rheumatology; b.i.d., twice daily; t.i.d., three times daily.

**Supplementary Figure S3.** Biomarkers (combined b.i.d. and t.i.d. dosing)

**(a)** Vectra DA score


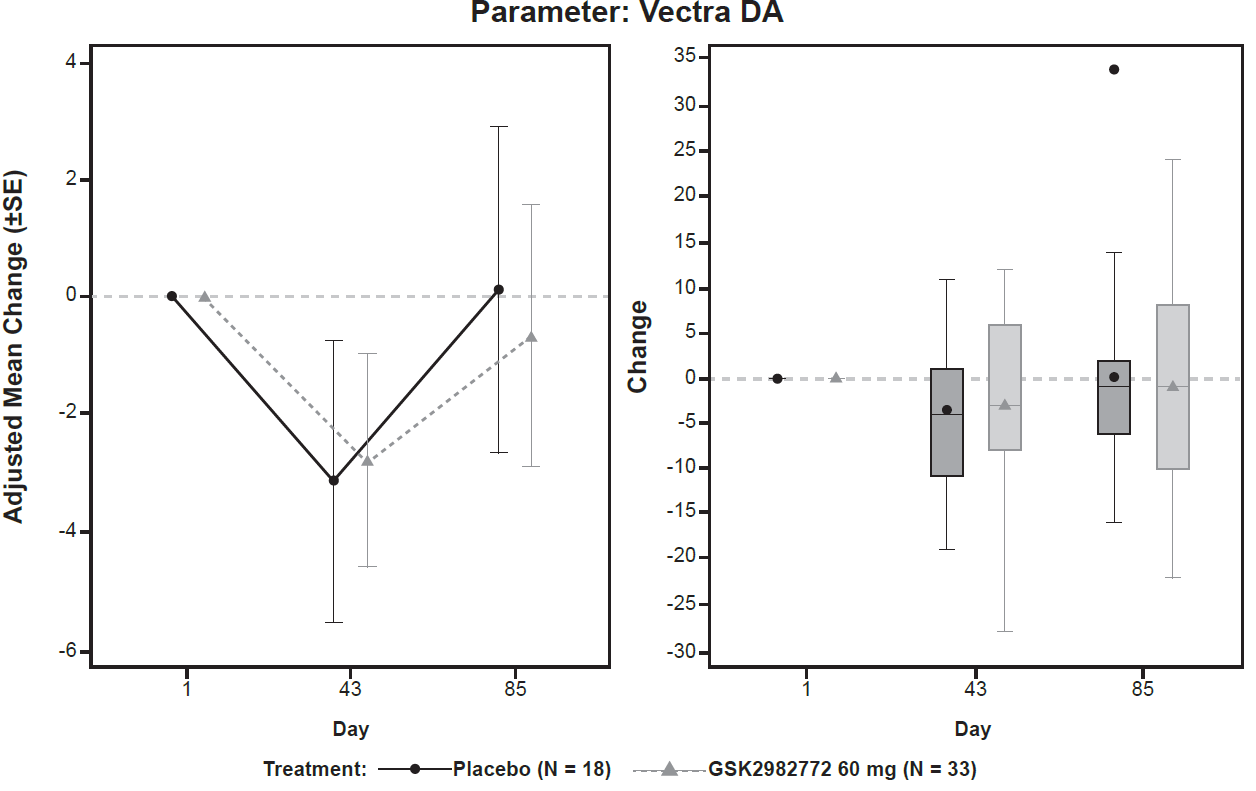


**(b)** MRP 8/14 (S100A8/S100A9)


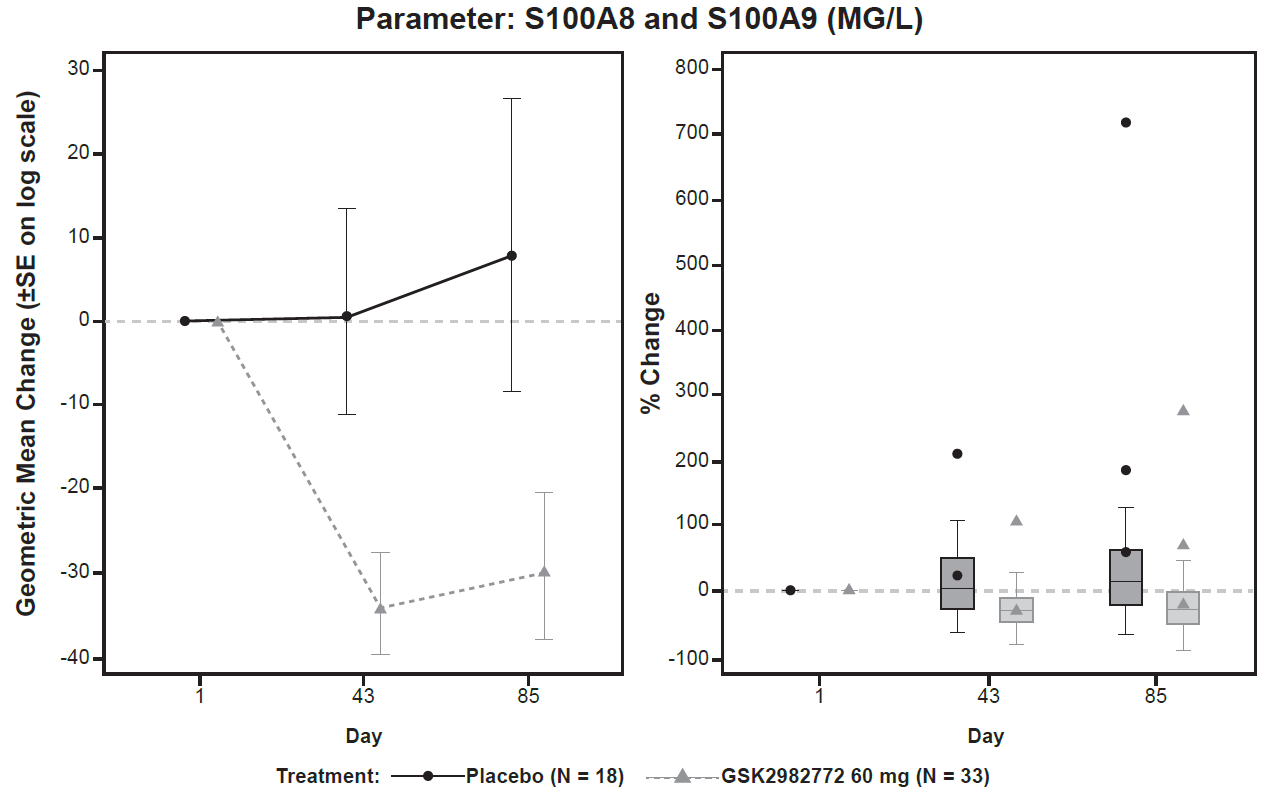


b.i.d., twice daily; MRP, Myeloid-related protein; t.i.d., three times daily.

**Supplementary Table S1.** Summary of inflammatory biomarkers in blood (combined b.i.d. and t.i.d. dosing)

| Biomarker  (Treatment, visit) | (n) | Mean | 95% CI for  (Lower, upper) | SD | Min | Max |
| --- | --- | --- | --- | --- | --- | --- |
| Vectra DA and components | | | | | | |
| Vectra DA |  |  |  |  |  |  |
| Placebo, day 1 | 18 | 50.8 | (43.4, 58.2) | 14.85 | 21 | 69 |
| Placebo, day 43 | 17 | 47.8 | (39.5, 56.0) | 16.06 | 17 | 72 |
| Placebo, day 85 | 17 | 51.1 | (42.0, 60.1) | 17.62 | 14 | 81 |
| GSK2982772, day 1 | 33 | 50.9 | (46.2, 55.7) | 13.39 | 29 | 89 |
| GSK2982772, day 43 | 29 | 48.3 | (42.8, 53.9) | 14.64 | 21 | 84 |
| GSK2982772, day 85 | 27 | 49.6 | (44.3, 55.0) | 13.45 | 19 | 78 |
| CRP (mg/L)^a^ |  |  |  |  |  |  |
| Placebo, day 1 | 18 | 28.2 | (15.2, 41.2) | 26.17 | 1 | 91 |
| Placebo, day 43 | 17 | 16.9 | (8.9, 24.8) | 15.41 | 1 | 52 |
| Placebo, day 85 | 17 | 28.3 | (10.0, 46.6) | 35.60 | 0 | 130 |
| GSK2982772, day 1 | 33 | 22.5 | (8.4, 36.6) | 39.83 | 1 | 207 |
| GSK2982772, day 43 | 29 | 17.8 | (8.2, 27.5) | 25.32 | 0 | 103 |
| GSK2982772, day 85 | 27 | 24.4 | (8.1, 40.6) | 41.13 | 0 | 165 |
| C3L1 (ng/L)^a^ |  |  |  |  |  |  |
| Placebo, day 1 | 18 | 118,896.8 | (56,295.0, 181,498.5) | 125,886.26 | 28,630 | 52,1895 |
| Placebo, day 43 | 17 | 139,433.4 | (65,832.7, 213,034.0) | 143,149.50 | 26,539 | 507,210 |
| Placebo, day 85 | 17 | 159,743.1 | (53,953.6, 265,532.5) | 205,755.00 | 29,633 | 910,535 |
| GSK2982772, day 1 | 33 | 91,947.8 | (71,488.7, 112,407.0) | 57,698.90 | 22,406 | 259,194 |
| GSK2982772, day 43 | 29 | 95,434.5 | (67,947.3, 122,921.7) | 72,262.50 | 27,205 | 381,833 |
| GSK2982772, day 85 | 27 | 88,894.6 | (63,242.8, 114,546.3) | 64,844.79 | 22,152 | 274,177 |
| EGF (ng/L)^a^ |  |  |  |  |  |  |
| Placebo, day 1 | 18 | 175.03 | (116.46, 233.60) | 117.783 | 15.7 | 369.8 |
| Placebo, day 43 | 17 | 179.31 | (104.83, 253.79) | 144.865 | 29.3 | 498.9 |
| Placebo, day 85 | 17 | 113.32 | (75.05, 151.60) | 74.442 | 8.7 | 264.3 |
| GSK2982772, day 1 | 33 | 173.90 | (132.75, 215.04) | 116.040 | 14.1 | 422.3 |
| GSK2982772, day 43 | 29 | 156.07 | (109.21, 202.92) | 123.181 | 9.8 | 505.2 |
| GSK2982772, day 85 | 27 | 124.14 | (88.44, 159.84) | 90.250 | 8.7 | 299.3 |
| IL6 (ng/L)^a^ |  |  |  |  |  |  |
| Placebo, day 1 | 18 | 64.42 | (24.36, 104.48) | 80.561 | 9.4 | 269.6 |
| Placebo, day 43 | 17 | 93.53 | (21.11, 165.95) | 140.845 | 4.0 | 481.4 |
| Placebo, day 85 | 17 | 89.11 | (13.00, 165.23) | 148.040 | 3.8 | 568.7 |
| GSK2982772, day 1 | 33 | 98.47 | (24.15, 172.78) | 209.577 | 6.6 | 1169.2 |
| GSK2982772, day 43 | 29 | 133.62 | (-14.14, 281.39) | 388.474 | 3.9 | 2045.7 |
| GSK2982772, day 85 | 27 | 50.59 | (24.17, 77.02) | 66.795 | 5.2 | 318.6 |
| Leptin (ng/L)^a^ |  |  |  |  |  |  |
| Placebo, day 1 | 18 | 23,647.5 | (11,005.6, 36289.4) | 25,421.60 | 1,880 | 88,224 |
| Placebo, day 43 | 17 | 23,157.6 | (12,909.9, 33,405.4) | 19,931.27 | 1,376 | 64,231 |
| Placebo, day 85 | 17 | 25,535.5 | (12,462.7, 38,608.2) | 25,425.80 | 1,793 | 89,439 |
| GSK2982772, day 1 | 33 | 19,157.7 | (14,465.7, 23,849.6) | 13,232.24 | 830 | 46,359 |
| GSK2982772, day 43 | 29 | 19,651.4 | (14,585.6, 24,717.2) | 13,317.76 | 857 | 47,513 |
| GSK2982772, day 85 | 27 | 21,246.3 | (14,464.7, 28,027.9) | 17,143.15 | 884 | 60,551 |
| MMP1 (ng/L)^a^ |  |  |  |  |  |  |
| Placebo, day 1 | 18 | 14,956.1 | (10,171.7, 19,740.6) | 9,621.10 | 4,092 | 36,495 |
| Placebo, day 43 | 17 | 12,455.0 | (8,954.0, 15,956.0) | 6,809.32 | 4,893 | 23,310 |
| Placebo, day 85 | 17 | 12,846.9 | (9,428.4, 16,265.3) | 6,648.72 | 5,821 | 28,585 |
| GSK2982772, day 1 | 33 | 9,912.7 | (7,842.5, 11,983.0) | 5,838.54 | 1,685 | 27,727 |
| GSK2982772, day 43 | 29 | 8,532.1 | (6,863.9, 10,200.3) | 4,385.52 | 1,800 | 16,888 |
| GSK2982772, day 85 | 27 | 9,240.2 | (7,022.2, 11,458.2) | 5,606.81 | 2,349 | 23,599 |
| MMP3 (ng/L)^a^ |  |  |  |  |  |  |
| Placebo, day 1 | 18 | 14,956.1 | (10,171.7, 19,740.6) | 9,621.10 | 4,092 | 36,495 |
| Placebo, day 43 | 17 | 12,455.0 | (8,954.0, 15,956.0) | 6,809.32 | 4,893 | 23,310 |
| Placebo, day 85 | 17 | 12,846.9 | (9,428.4, 16,265.3) | 6,648.72 | 5,821 | 28,585 |
| GSK2982772, day 1 | 33 | 9,912.7 | (7,842.5, 11,983.0) | 5,838.54 | 1,685 | 27,727 |
| GSK2982772, day 43 | 29 | 8,532.1 | (6,863.9, 10200.3) | 4,385.52 | 1,800 | 16,888 |
| GSK2982772, day 85 | 27 | 9,240.2 | (7,022.2, 11,458.2) | 5,606.81 | 2,349 | 23,599 |
| Resistin (ng/L)^a^ |  |  |  |  |  |  |
| Placebo, day 1 | 18 | 8,114.9 | (6,613.2, 9,616.6) | 3,019.82 | 3,588 | 16,302 |
| Placebo, day 43 | 17 | 7,815.3 | (6,599.8, 9,030.8) | 2,364.07 | 4,583 | 12,672 |
| Placebo, day 85 | 17 | 8,186.8 | (6,845.0, 9,528.5) | 2,609.59 | 5,181 | 15,054 |
| GSK2982772, day 1 | 33 | 8,390.5 | (7,344.0, 9,437.1) | 2,951.40 | 3,612 | 20,844 |
| GSK2982772, day 43 | 29 | 7,353.4 | (6,458.7, 8,248.1) | 2,352.12 | 4,283 | 16,019 |
| GSK2982772, day 85 | 27 | 7,906.8 | (6,668.5, 9,145.1) | 3,130.23 | 4,007 | 21,352 |
| Serum amyloid A (ng/L)^a^ |  |  |  |  |  |  |
| Placebo, day 1 | 18 | 12,098,768.9 | (4,230,683.7, 19,966,854.2) | 15,821,979.37 | 381,497 | 62,204,569 |
| Placebo, day 43 | 17 | 7,470,845.1 | (2,866,737.2, 12,074,953.0) | 8,954,750.55 | 211,381 | 25,880,514 |
| Placebo, day 85 | 17 | 15,829,562.9 | (465,530.1, 31,193,595.7) | 29,882,245.20 | 278,896 | 11,833,3485 |
| GSK2982772, day 1 | 33 | 15,701,622.2 | (5,646,385.4, 25,756,858.9) | 28,357,794.59 | 546,520 | 132,933,322 |
| GSK2982772, day 43 | 29 | 12,621,637.8 | (3,461,933.1, 21,781,342.5) | 24,080,427.32 | 873,883 | 100,012,482 |
| GSK2982772, day 85 | 27 | 19,321,651.6 | (6,827,566.8, 31,815,736.5) | 31,583,672.88 | 680,558 | 115,671,570 |
| TNFR1 (ng/L)^a^ |  |  |  |  |  |  |
| Placebo, day 1 | 18 | 1,696.1 | (1,328.5, 2,063.7) | 739.25 | 648 | 3,485 |
| Placebo, day 43 | 17 | 1,689.4 | (1,340.8, 2,038.0) | 678.04 | 601 | 3,175 |
| Placebo, day 85 | 17 | 1,762.7 | (1,319.2, 2,206.2) | 862.65 | 495 | 3,537 |
| GSK2982772, day 1 | 33 | 1,464.7 | (1,351.6, 1,577.8) | 319.01 | 902 | 2,063 |
| GSK2982772, day 43 | 29 | 1,397.3 | (1,267.6, 1,527.0) | 340.88 | 856 | 2,097 |
| GSK2982772, day 85 | 27 | 1,429.5 | (1,280.5, 1,578.6) | 376.77 | 730 | 2,317 |
| VCAM1 (ng/L)^a^ |  |  |  |  |  |  |
| Placebo, day 1 | 18 | 743,334.4 | (645,881.5, 840,787.3) | 195,968.64 | 453,191 | 1,214,951 |
| Placebo, day 43 | 17 | 716,461.5 | (604,250.5, 828,672.6) | 218,244.70 | 461,965 | 1,196,486 |
| Placebo, day 85 | 17 | 757,292.5 | (614,299.8, 900,285.3) | 278,113.48 | 482,508 | 1,325,424 |
| GSK2982772, day 1 | 33 | 650,749.9 | (606,738.1, 694,761.8) | 124,122.20 | 430,579 | 985,518 |
| GSK2982772, day 43 | 29 | 652,691.5 | (600,982.1, 704,400.9) | 135,941.56 | 412,867 | 1,020,467 |
| GSK2982772, day 85 | 27 | 675,912.6 | (615,411.7, 736,413.6) | 152,939.74 | 416,442 | 1,005,030 |
| VEGF (ng/L)^a^ |  |  |  |  |  |  |
| Placebo, day 1 | 18 | 253.34 | (180.72, 325.97) | 146.050 | 114.3 | 698.0 |
| Placebo, day 43 | 17 | 252.59 | (179.96, 325.22) | 141.256 | 127.4 | 635.0 |
| Placebo, day 85 | 17 | 254.56 | (185.83, 323.30) | 133.688 | 91.4 | 583.1 |
| GSK2982772, day 1 | 33 | 246.49 | (204.89, 288.09) | 117.320 | 105.4 | 518.5 |
| GSK2982772, day 43 | 29 | 238.60 | (194.32, 282.88) | 116.412 | 78.8 | 499.4 |
| GSK2982772, day 85 | 27 | 246.76 | (195.90, 297.62) | 128.570 | 94.8 | 611.2 |
| Other biomarkers | | | | | | |
| MCP1 (ng/L) |  |  |  |  |  |  |
| Placebo, day 1 | 18 | 837.811 | (402.408, 1,273.214) | 875.5548 | 76.12 | 3,303.59 |
| Placebo, day 43 | 17 | 1,117.072 | (782.087, 1,452.058) | 651.5288 | 64.34 | 2,513.69 |
| Placebo, day 85 | 17 | 1,172.659 | (764.077, 1,581.241) | 794.6713 | 64.16 | 3,345.90 |
| GSK2982772, day 1 | 33 | 924.264 | (658.482, 1,190.045) | 749.5577 | 67.04 | 3,105.86 |
| GSK2982772, day 43 | 28 | 1,265.531 | (1,001.924, 1,529.137) | 679.8198 | 99.99 | 2,639.46 |
| GSK2982772, day 85 | 26 | 1,278.709 | (955.410, 1,602.009) | 800.4266 | 61.73 | 4,188.58 |
| MMIF (µg /L) |  |  |  |  |  |  |
| Placebo, day 1 | 18 | 27.541 | (21.076, 34.006) | 13.0004 | 14.10 | 57.07 |
| Placebo, day 43 | 17 | 27.323 | (20.947, 33.698) | 12.4001 | 11.88 | 52.95 |
| Placebo, day 85 | 17 | 22.141 | (17.434, 26.848) | 9.1554 | 12.39 | 42.02 |
| GSK2982772, day 1 | 33 | 40.562 | (21.378, 59.747) | 54.1037 | 11.97 | 277.24 |
| GSK2982772, day 43 | 29 | 39.802 | (20.241, 59.363) | 51.4247 | 11.00 | 249.19 |
| GSK2982772, day 85 | 27 | 23.278 | (20.186, 26.369) | 7.8147 | 12.79 | 43.42 |
| MMP13 (ng/L) |  |  |  |  |  |  |
| Placebo, day 1 | 16 | 562.516 | (67.860, 1,057.171) | 928.2983 | 31.25 | 3,552.37 |
| Placebo, day 43 | 16 | 534.231 | (19.079, 1,049.382) | 966.7631 | 31.25 | 3,544.50 |
| Placebo, day 85 | 14 | 706.756 | (54.877, 1,358.636) | 1,129.0246 | 31.25 | 3,625.83 |
| GSK2982772, day 1 | 31 | 1,074.827 | (541.371, 1,608.283) | 1,454.3402 | 31.25 | 5,630.40 |
| GSK2982772, day 43 | 27 | 1,041.618 | (486.326, 1,596.911) | 1,403.7183 | 31.25 | 4,269.36 |
| GSK2982772, day 85 | 23 | 1,117.440 | (511.818, 1,723.063) | 1,400.5030 | 31.25 | 4,363.55 |
| S100A8 and S100A9  (MRP8/14) (mg/L) |  |  |  |  |  |  |
| Placebo, day 1 | 18 | 5.927 | (3.563, 8.292) | 4.7552 | 1.24 | 16.70 |
| Placebo, day 43 | 17 | 6.162 | (3.738, 8.586) | 4.7142 | 0.90 | 15.73 |
| Placebo, day 85 | 17 | 6.825 | (4.172, 9.478) | 5.1601 | 0.77 | 17.32 |
| GSK2982772, day 1 | 33 | 6.975 | (5.051, 8.899) | 5.4268 | 1.13 | 23.15 |
| GSK2982772, day 43 | 29 | 4.521 | (2.987, 6.056) | 4.0336 | 1.12 | 19.83 |
| GSK2982772, day 85 | 27 | 4.207 | (3.033, 5.380) | 2.9660 | 0.78 | 15.31 |
| TIMP1 (µg/L) |  |  |  |  |  |  |
| Placebo, day 1 | 18 | 354.2381 | (284.7852, 423.6910) | 139.66318 | 172.405 | 755.833 |
| Placebo, day 43 | 17 | 299.0171 | (251.8729, 346.1612) | 91.69294 | 156.206 | 477.269 |
| Placebo, day 85 | 17 | 341.9860 | (252.6108, 431.3612) | 173.83021 | 126.643 | 755.566 |
| GSK2982772, day 1 | 33 | 319.1482 | (271.9443, 366.3521) | 133.12445 | 129.071 | 722.526 |
| GSK2982772, day 43 | 29 | 275.9791 | (239.1022, 312.8559) | 96.94742 | 128.029 | 598.606 |
| GSK2982772, day 85 | 27 | 284.0283 | (248.0056, 320.0510) | 91.06141 | 152.257 | 540.011 |

^a^Part of the Vectra DA score.

b.i.d., twice daily; C3L1, chitinase 3 like-1; CRP, C-reactive protein; EGF, epidermal growth factor; IL6, interleukin 6; MCP1, monocyte chemotactic protein; MMIF, macrophage migration inhibitory factor; MMP1, matrix metalloproteinase 1; MMP3, matrix metalloproteinase 3; MMP13, matrix metalloproteinase 13; MRP, Myeloid-related protein; t.i.d., three times daily; TIMP1, tissue inhibitor of metalloproteinase 1; TNFR1, tumor necrosis factor receptor type 1; VCAM1, vascular cell adhesion molecule 1; VEGF, vascular endothelial growth factor.
